# Kinin B1 receptor antagonism is equally efficient as angiotensin receptor 1 antagonism in reducing renal fibrosis in experimental obstructive nephropathy, but is not additive

**DOI:** 10.3389/fphar.2015.00008

**Published:** 2015-02-02

**Authors:** Antoine Huart, Julie Klein, Julien Gonzalez, Bénédicte Buffin-Meyer, Eric Neau, Christine Delage, Denis Calise, David Ribes, Joost P. Schanstra, Jean-Loup Bascands

**Affiliations:** ^1^Institut National de la Santé et de la Recherche Médicale U1048, Institute of Cardiovascular and Metabolic DiseaseToulouse, France; ^2^Department of Nephrology, CHU-RangueilToulouse, France; ^3^Université Toulouse III Paul-SabatierToulouse, France; ^4^Unité mixte de Service US006, CHU-RangueilToulouse, France

**Keywords:** angiotensin receptor inhibition, bradykinin B1 receptor, combined therapy, low density array, renal fibrosis

## Abstract

**Background:** Renal tubulointerstitial fibrosis is the pathological hallmark of chronic kidney disease (CKD). Currently, inhibitors of the renin–angiotensin system (RAS) remain the sole therapy in human displaying antifibrotic properties. Further antifibrotic molecules are needed. We have recently reported that the delayed blockade of the bradykinin B1 receptor (B1R) reduced the development of fibrosis in two animal models of renal fibrosis. The usefulness of new drugs also resides in outperforming the gold standards and eventually being additive or complementary to existing therapies.

**Methods:** In this study we compared the efficacy of a B1R antagonist (B1Ra) with that of an angiotensin type 1 receptor antagonist (AT1a) in the unilateral ureteral obstruction (UUO) model of renal fibrosis and determined whether bi-therapy presented higher efficacy than any of the drugs alone.

**Results:** B1R antagonism was as efficient as the gold-standard AT1a treatment. However, bitherapy did not improve the antifibrotic effects at the protein level. We sought for the reason of the absence of this additive effect by studying the expression of a panel of genes involved in the fibrotic process. Interestingly, at the molecular level the different drugs targeted different players of fibrosis that, however, in this severe model did not result in improved reduction of fibrosis at the protein level.

**Conclusions:** As the B1R is induced specifically in the diseased organ and thus potentially displays low side effects it might be an interesting alternative in cases of poor tolerability to RAS inhibitors.

## Introduction

The incidence and prevalence of chronic kidney disease (CKD) is increasing worldwide, largely due to the increasing incidence of type 2 diabetes and obesity (El Nahas, 2005; Vilayur and Harris, 2009; Levey and Coresh, 2012). Most patients with CKD progress toward end-stage renal disease (ESRD) within 10–30 years requiring renal replacement therapies. In addition to evolution toward ESRD, CKD is now recognized as a major risk factor for cardiovascular disease since patients with CKD are far more likely to die from cardiovascular pathologies than to develop ESRD (Keith et al., 2004). Delaying the progression of CKD will therefore not only reduce the number of patients ending up with ESRD, but also the number of patients with severe cardiovascular complications.

Renal tubulointerstitial fibrosis is the pathological hallmark of CKD. Although the initial renal disease leading to CKD can be different, the mechanisms leading to renal fibrosis are thought to be similar. Briefly, renal cell injury leads to the synthesis and secretion of cytokines and chemokines. In response to these inflammatory mediators, mononuclear cells progressively infiltrate the interstitial space and differentiate into macrophages. Macrophages perpetuate inflammation, leading to the proliferation of myofibroblasts, the cells responsible for the secretion of soluble pro-fibrotic molecules including growth factors, cytokines, chemokines, and extracellular matrix (ECM) proteins that contribute to progression of renal fibrosis. For in-depth reviews please refer to the following articles (Harris and Neilson, 2006; Duffield, 2014).

A multitude of events and factors were identified to be involved in the development of renal fibrosis, potentially leading to new antifibrotic strategies and compounds (Strutz, 2001; Iwano and Neilson, 2004; Vilayur and Harris, 2009). However, in humans, blockade of the renin–angiotensin system (RAS) remains the only effective therapy (Vilayur and Harris, 2009). Thus, any new potential anti-fibrotic therapy should be compared to angiotensin converting enzyme inhibition (ACEi) or AT1 receptor antagonists (AT1a). In addition, each new drug should be tested in combination with RAS inhibitors to determine the efficacy as an “add-on” drug.

We have reported (Klein et al., 2009) that the blockade of the bradykinin B1 receptor (B1R) was associated to a curative antifibrotic effect in the unilateral ureteral obstruction (UUO) model as well as in a model of glomerulonephritis (Klein et al., 2010).

The aim of the present study was to compare the antifibrotic potential of a B1R antagonist (B1Ra) to an AT1a in the UUO model and to investigate whether association of both compounds results in additive antifibrotic effects compared to each drug individually. Drugs were administrated at day 3 post-UUO to evaluate a curative effect.

We found that B1R antagonism was as efficient as the gold-standard AT1 antagonism. Bitherapy did not improve the antifibrotic effects histologically. However, we observed that, at the gene expression level, whereas each drug alone down-regulated significantly 29 and 17 genes (for AT1a and B1Ra, respectively), bitherapy largely increased this number of down regulated genes.

## Subjects and methods

### Drugs

B1Ra SSR240612 was synthesized at Sanofi-Aventis R&D Montpellier-France (Gougat et al., 2004). The SSR240612 solution was administered by gavage at a dose of 10 mg/kg/d as described previously (Klein et al., 2010). AT1a (Valsartan was purchased from Novartis) was administrated by gavage at a dose of 30 mg/kg/d.

### Animals

We used C57Bl/6J mice (Harlan). The mice were housed in a pathogen-free environment. All experiments reported were conducted in accordance with the NIH guide for the care and use of laboratory animals and were approved by a local animal care and use committee (CEEA-122 2014-06).

Treatments with the AT1a and B1Ra were started 3 days after ureteral obstruction surgery and continued throughout the time (8 days) of obstruction. The control group received one hundred microliter of water by gavage. At the end of the different protocols, mice were sacrificed, and the kidneys were removed and divided in different parts according to the different protocols employed.

### Unilateral ureteral obstruction (UUO)

Male mice of 8 weeks of age were used for these experiments. The unilateral ureteral ligation was performed as previously described (Schanstra et al., 2002; Klein et al., 2009). Briefly, under oxygen-isoflurane anesthesia and through a longitudinal, left abdominal incision, the ureter was exposed and ligated with a 6/0 nylon thread at the uretero-pelvic junction. In sham operations, the ureter was exposed but not ligated and repositioned. Mice were maintained on a standard mouse chow and tap water.

### Immunohistochemistry and histological analysis

From paraffin-embedded kidney sections routine histology and immunohistological staining and analysis were performed as previously described (Schanstra et al., 2002; Pradere et al., 2007; Klein et al., 2009, 2010). Three- to four-micrometer sections were cut and used for routine staining (hematoxylin–eosin and Sirius red staining) and immunohistochemistry. For immunohistochemistry, mouse renal tissue were first de-waxed in toluene and rehydrated through a series of graded ethanol washes before endogenous peroxidase blockage. Specific primary antibodies were incubated (1 h at room temperature) on mouse kidney sections for the detection of collagen type III (1/500) (Interchim), an extracellular matrix protein, or F4/80 (1/250) (RM2900; Caltag laboratories Inc., Burlingame, California, USA), as a marker of macrophages. For visualization we used the Dako Envision system. Sections were finally counterstained with hematoxylin. Negative controls for the immunohistochemical procedures included substitution of the primary antibody with non-immune sera.

Histomorphometric analyses were performed as recently described (Klein et al., 2009) using commercially available image-analysis software that allows rebuilding of a kidney section from adjacent individual captures (Explora Nova Mosaïc software, La Rochelle, France). The number of colored pixel (red or brown) was determined, by blinded analysis. Results are expressed as percentage of specific colored pixel/total number of pixel analyzed.

### RNA extraction and reverse transcription

Total RNA was purified from frozen renal tissues using the Qiagen RNeasy® Mini Kit following the manufacturer's protocol and digested with TURBO DNase™ (Ambion).

Total RNA was quantified by Nanodrop ND-1000 and the quality was checked using an Experion Automated Electrophoresis Station with a RNA StdSens Analysis Kit and Experion version 2.0 software (Bio-Rad). Ten micrograms of total RNA were transcribed into cDNA in a total volume of 100 μl using the High Capacity cDNA Archive Kit and performed according the manufacturer's instructions (Applied Biosystems).

### TaqMan low density array (TLDA)

The LDA contains eight samples-loading lines, each connected by microchannel to 48 miniature reaction chambers for a total of 384 wells per card. We created a ≪fibrosis≫ taqMan Low-density array based on Applied biosystems 7900HT Microfluidic card. Gene-specific primers and FAM-labeled probes (Assays-on-Demand, Applied Biosystems) were lyophilized in each well. We choose 96 genes (Supplementary Table 1): 92 fibrosis-related genes plus four housekeeping genes (Gapdh, Hmbs, Hprt, 18S) and configured our LDA cards with four identical 96 genes sets (two samples in duplicate). The four reference genes were chosen among 16 that we have tested for their relative stability expression. Indeed, it has been clearly shown that that normalization against a single reference gene is not any more acceptable (Murphy and Bustin, 2009). To reduce the risk of false interpretation of the gene expression variation, it is necessary to have a optimize normalization with several stable reference genes. To this end we have evaluated by qPCR the expression of 16 reference genes in our different experimental conditions. We used the free geNorm software to test the stability of 16 reference genes in normal and fibrotic conditions in presence or not of the different drugs. As shown in Supplementary Figure 1, the most stable genes in our different experimental conditions were Gapdh and Hmbs.

One Hundred nanograms of cDNA dissolved in 1X TaqMan Universal PCR MasterMix (Applied Biosystems) were added to each line of the array and the reaction was performed on ABI 7900 HT Fast Real-Time PCR System (Applied Biosystems). The PCR conditions were as follows: 2 min at 50°C, 10 min at 94.5°C, 40 cycles of denaturation at 97°C for 30 s, and annealing and extension at 59.7°C for 1 min. The quantitative cycle Cq was automatically given by SDS 2.2 software package (Applied Biosystems). The relative amount of each gene mRNA to the mean of the two housekeeping genes (Gapdh and Hmbs) was calculated as 2^−Δ*Cq*^ where Δ*Cq* = *Cq*_gene_ − *Cq*_mean of housekeeping genes_. The fold-change of each gene mRNA to the normal condition was calculated as 2^−ΔΔ*Cq*^ where ΔΔ*Cq* = Δ*Cq*_gene in fibrotic condition_ − Δ*Cq*_gene in normal condition_.

### Semiquantitative RT-PCR

Low density array data validation was performed for ten selected genes as described previously using the Gapdh as housekeeping gene (Bascands et al., 2009).

### Statistical analysis

Data are expressed as mean plus or minus SD. ANOVA with *post-hoc* Tuckey α-test was performed for comparison between the different groups. *p*-values less than 0.05 were considered statistically significant.

## Results

### Effect of delayed administration of AT1 and B1R antagonists alone or in combination on renal tissue histology

**Figure 1:**
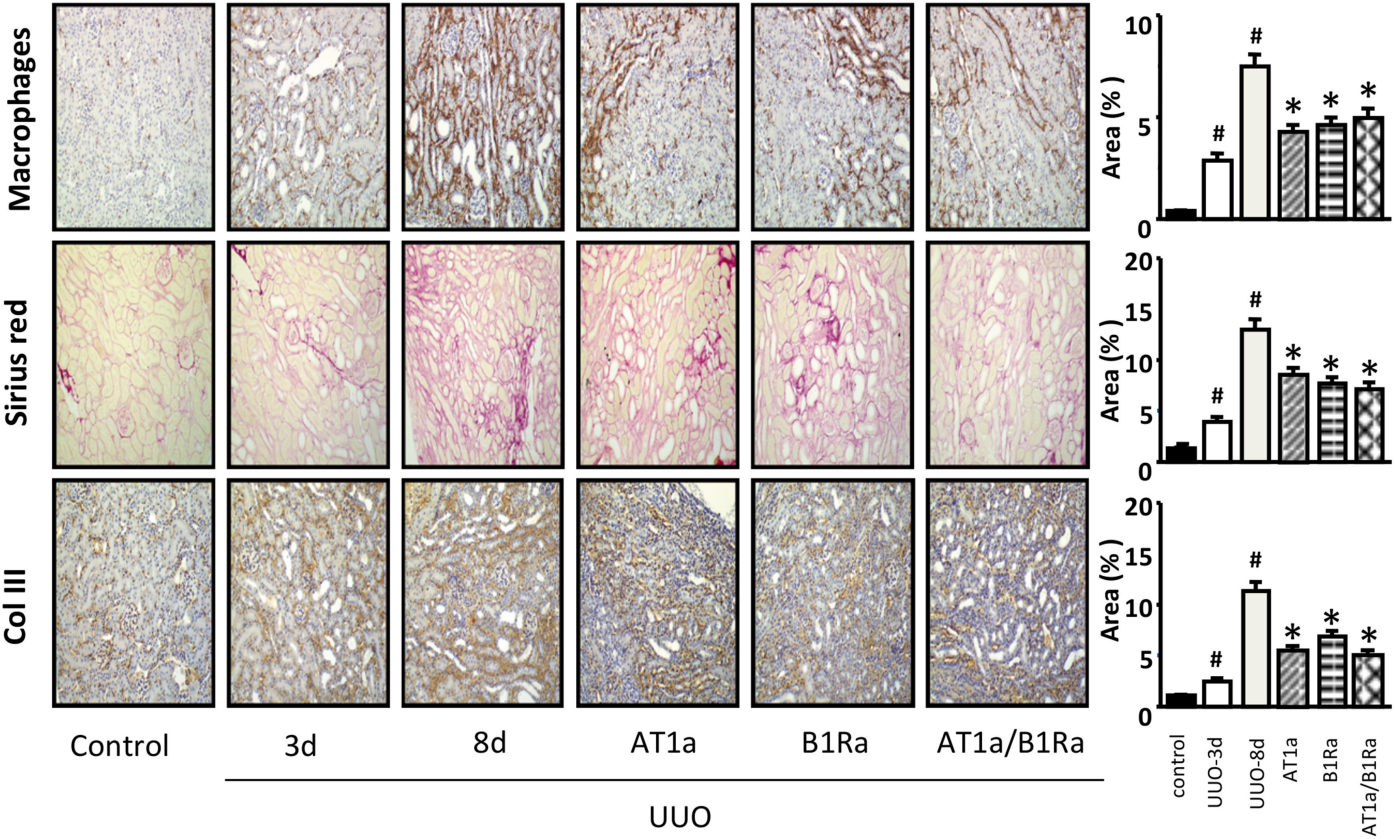
**Accumulation of macrophages, extracellular matrix (Sirius red) and collagen type III in obstructed kidneys 3 and 8 days after UUO and protective effect induced by administration of the AT1a, B1Ra or both starting at day 3 following ureteral obstruction surgery**. Histograms represent the semi quantification of the immunohistological staining. *N* = 8/group. ^#^*P* < 0.05 vs. control and ^*^P < 0.05 vs. UUO-8 days.

As expected, from the third day to the eight day after UUO we observed a progressive increase in macrophage infiltration and interstitial fibrosis (Figure 1). Both AT1a and B1Ra prevented significantly and at the same level the accumulation of macrophages and ECM. Co-administration of the two antagonists did not show an additional protective effect on the measured parameters.

### Profiling of genes involved in fibrosis

**Figure 2:**
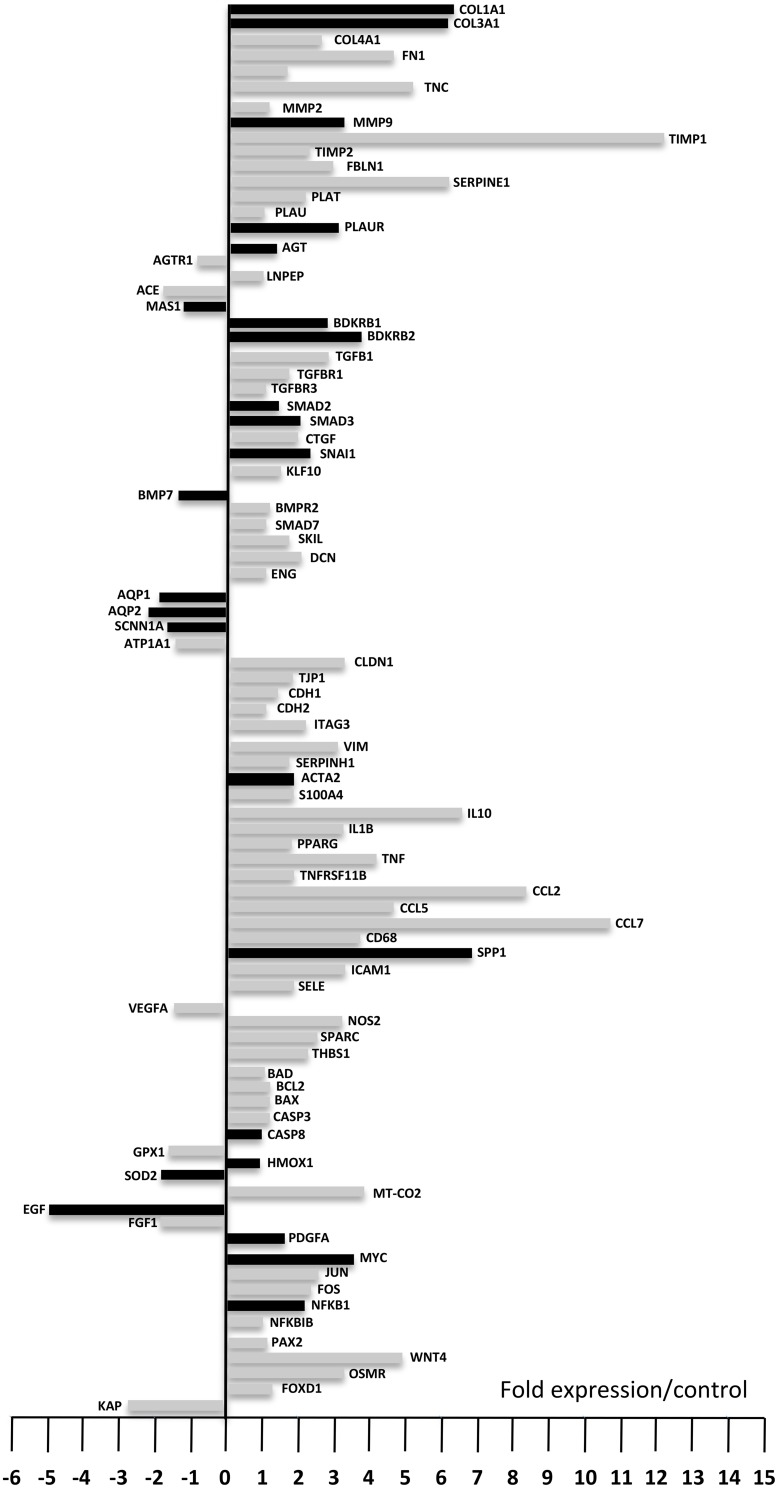
**Gene expression levels after 8 days of UUO compared to control**. Down-regulated genes are displayed by the bars on the left while up-regulated genes are displayed on the right (fold expression compared to control). Black bars represent genes that already displayed up- or down-regulation 3 days after UUO. Each bar represents the mean from eight animals. Fold expression between −1 and +1 was considered not significant.

To better understand the molecular mechanisms involved in the development of UUO-induced fibrosis and in the response to AT1 and B1R antagonists we performed expression profiling of genes involved in the development of fibrosis. Figure 2 represents the expression variations of 87 genes out of the 93 studied since five genes (Ren2, Agtr2, Klklb1, Igf1, and Il6 which are highlighted in blue in Supplementary Table 1) were not detected in our conditions, due to either a very low expression level or a poor primer efficacy.

Among these 87 genes nine genes (Plau, Agtr1, Lnpep (IRAP), Tgfbr3, Smad7, Cdh2, Bad, Nfkbib, and Pax2) did not show significant variation after UUO, 13 genes were significantly down-regulated and 65 genes were significantly up-regulated after 8 days UUO. Interestingly 17 out of the 65 up-regulated genes, and seven of the 13 down-regulated genes were already modified 3 days after UUO (Figure 2, black bars on the right and left-hand side, respectively). These variations were validated by semi-quantitative RT-PCR on 10 arbitrarily selected genes (Table 1).

**Table 1:** **RT-PCR validation of TaqMan Low Density Array (TLDA)**.

**Gene name**	**Fold expression-TLDA**	**Control RT-PCR**
Col3a1	+6.27	+3.97
Timp1	+12.34	+22.75
Plaur	+3.16	+5.16
Bdkrb1	+2.97	+3.24
Tgfb1	+2.95	+6.13
Bmp7	−1.33	−1.67
Ccl7	+10.89	+36.21
Nos2	+3.22	+7.07
Mt-Co2	+4.96	+12.55
Egf	−5.02	−3.76

*RT-PCR values represent medium value of eight RT-PCR experiments*.

### Gene expression variations induced by delayed administration of AT1 and B1R antagonists alone or in combination

Table 2 shows *p*-values for genes expression differences in animals treated by AT1a or B1Ra or both antagonists after 8 days of UUO. In total, 29 genes were down-regulated by AT1a, 17 by B1Ra and 54 by the combined treatment. Interestingly, 25 genes (highlighted in yellow) were not significantly down-regulated by the antagonists separately, however, the co-administration of both antagonists led to the significant down regulation of these genes. Moreover, whereas 15 genes (highlighted in green) and three genes (highlighted in orange) were specifically down-regulated by only one treatment (AT1a and B1Ra, respectively), combined treatment amplified down-regulation of 14 of these 18 genes as shown by the increased *p*-value (last column, Table 2). This effect was particularly marked for Ctgf (highlighted in blue). Conversely although the B1Ra significantly down regulated Snai1, the addition of the AT1a led to a non-significant *p*-value. Similarly Wnt 4 and Nos2 were significantly down regulated by the AT1a and became non-significant by the co-administration of the B1Ra.

**Table 2:**
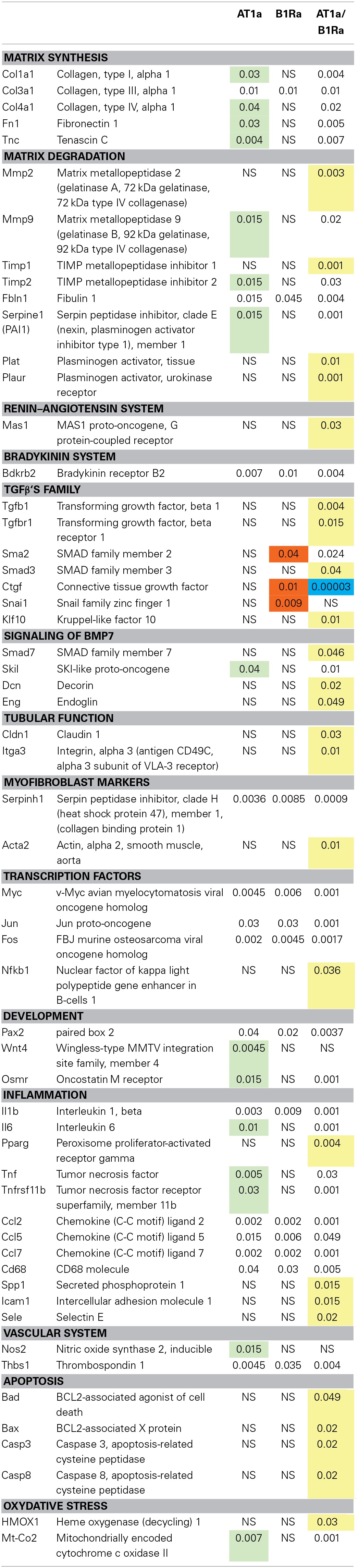
**Down regulated genes 8 days after UUO by either angiotensin type 1 receptor antagonist (AT1a), bradykinin type 1 receptor antagonist (B1Ra) or both AT1a/B1Ra treatments**.

*Each value represents the p-value, NS, Non-Significant. Green boxes represent genes exclusively down regulated by AT1a. Orange boxes represent genes exclusively down regulated by the B1Ra. Yellow boxes represent genes exclusively down regulated by co-administration of AT1a and B1Ra. The additive effect of co-treatment for CTGF gene expression is highlighted in blue*.

## Discussion

The data presented in this study are the first evidence that delayed treatment with a B1R antagonist is as efficient as AT1 receptor antagonism to slow down renal tubulointerstitial fibrosis. The results also demonstrate that in our experimental conditions, the effects of both compounds are not additive. Indeed we do not observe a significant improvement of renal tubulointerstitial fibrosis, assessed by (immuno)histochemistry in the animal group treated with both antagonists compared to the renal protective effect observed with each agent individually. To understand the underlying mechanisms of these observations we have studied the expression level of a number of genes known to be involved in the renal fibrosis process.

As already reported (Morrissey et al., 1996; Klahr, 2000; Chevalier et al., 2009; Duffield, 2014), we confirmed the up- or down-regulation of several genes involved in the renal fibrotic process following ureteral obstruction such as Tgfb1, Ctgf, collagens types (I, III, IV), fibronectin, Nfkb, growth factors, chemokines, etc… We also confirmed at the tissue level the antifibrotic effect of AT1a (Ishidoya et al., 1995) and B1Ra (Klein et al., 2009). At the gene level, 29 genes were modified by AT1a and 17 by B1Ra. Surprisingly, most of the genes down-regulated by the B1Ra were common to those modified by the AT1a treatment, as only three out of 17 were specifically down-regulated by the B1Ra. However, it is very interesting to point out that the B1Ra specifically down-regulated Snai1 and Ctgf. Indeed, these two genes are crucial in the renal fibrotic mechanism since they are key downstream effectors of the TGFβ pathway and consequently significantly involved in the appearance of myofibroblasts and the production of ECM (Qi et al., 2005; Yoshino et al., 2007).

Interestingly, although each antagonist individually was able to induce down-regulation of fibrotic genes, a clear synergistic effect of the combination of both antagonists was observed on the expression of a number of genes. This point is illustrated by the significant further down-regulation of Tnfrsf11b in presence of both antagonists. This effect on Tnfrsf11b is quite relevant since the HSP47 is clearly involved in renal fibrogenesis through its role in collagen biosynthesis (Razzaque et al., 2005). In addition, *in vivo*, the use of small interfering RNA against Tnfrsf11b mRNA significantly decreases interstitial collagen accumulation (Xia et al., 2008).

Similarly, co-administration of both antagonists significantly down-regulated 25 genes that were not modified by the drugs alone including the profibrotic gene Tgfb1 (Garcia-Sanchez et al., 2010) as well as nuclear factor Nfkb (Guijarro and Egido, 2001). The observed down-regulation of those two genes could in part explain the down-regulation of the other genes which are either under the direct control of NF-κB or stimulated by TGFβ1.

In addition we observed for around ten genes, mainly involved in extra-cellular matrix accumulation, an incremental nephroprotective effect by the addition of the B1Ra to the already well-known AT1a therapeutic effect. This effect was demonstrated by the increased *p*-values observed in the presence of both antagonists while for single administration, only the AT1a induced a significant down-regulation. Conversely whereas Smad2 and Ctgf were only down regulated by the B1Ra, the association of the AT1a led to a significant decrease in the *p*-value. This drop in the *p*-value was particularly impressive for Ctgf. The down-regulation of Ctgf by the B1Ra was not unexpected since we have previously reported this effect (Klein et al., 2009) in the UUO model and an *in-vitro* study has demonstrated that the B1R stimulation induced collagen type I synthesis via stabilization of Ctgf mRNA (Ricupero et al., 2000). However, we did not expect exacerbation of the effect by the combination with an AT1a. This strongly suggests that both inhibitors stimulate different pathways leading to the inhibition of Ctgf expression. Indeed it is well-known that Angiotensin II induces TGFβ1 expression (Wolf, 2006) and the induction of CTGF by TGFβ1 has been shown to be Smad3 and Smad4 dependent and Smad2 independent (Phanish et al., 2006). On the other hand we observed in the present study that the B1Ra induced an inhibition of Smad2 expression, which might represent an additional pathway in the regulation of Ctgf expression. On the contrary, as shown in Table 2, Snail1, Wnt4, and Nos2, which were significantly down-regulated either by the B1Ra or the AT1a became non-significantly down-regulated with the co-administration of both antagonists. Keeping in mind the role of Snail1 and Wnt4 in the fibrotic process (Surendran et al., 2002), one could expect an impact on the loss of the epithelial phenotype leading to an increased appearance of myofibroblasts, however the significant decrease in the expression of Tnfrsf11b and Acta2, two myofibroblast-markers, do not comfort this hypothesis. Regarding Nos2 mRNA expression, a non-significant down-regulation might be beneficial since it suggests increased NO production, which is well-known to be nephroprotective (Morrissey et al., 1996).

Finally despite these positive effects observed at the level of gene expression we observed the absence of a visible protective effect at the tissue level at least in our experimental conditions. We hypothesize that the absence of effect at the tissue level has to be related to both the UUO model and the curative protocol used. The UUO model has the drawback of its advantages, it is an accelerated and reproducible *in vivo* model of renal fibrosis and therefore easy to use in the laboratory, but is far from mimicking the timescale of progression of fibrotic CKD which in human evolves over years. Thus, although the UUO model is widely used to identify early profibrotic events, potential profibrotic protein/pathways as well as antifibrotic molecules (Bascands and Schanstra, 2005), this model is most likely too drastic and not enough progressive to appreciate histologically any significant additive therapeutic effect. Indeed, although recent studies using this UUO model have reported additive antifibrotic effect of various drugs such as paricalcitol (Tan et al., 2009), pioglitazone (Higashi et al., 2010), and Rho-kinase inhibitor (Takeda et al., 2010) with renin angiotensin-inhibitors, those beneficial additive effects were obtained by preventive treatment, whereas in our study we attempted to demonstrate a therapeutic effect by administrating the compounds 3 days after induction of disease.

In summary the most important result of the present study is that kinin B1 receptor antagonism is as efficient in reducing renal fibrosis as angiotensin receptor 1 antagonism in the UUO model using a curative treatment protocol. This point has to be kept in mind because (i) the B1R is induced specifically in the diseased organ and thus potentially displays low side effects and (ii) it might become an alternative therapy in cases of poor tolerability due to the known adverse effects (chronic cough, hyperkalemia, angio-edema) of angiotensin converting enzyme inhibitors or to a lesser extent of AT1 receptor antagonist.

If targeting the renin–angiotensin system is now a well-admitted therapy for CKD, our data strongly suggested that a combination therapy associating an AT1a and a B1Ra might be much more effective to slow down the progression of renal fibrosis. However, this combination has to be evaluated in more chronic model (such as subtotal nephrectomy, glomerulonephritis, diabetic nephropathy) of renal disease associated to the slow progression of renal fibrosis.

## Author contributions

Antoine Huart, Julie Klein, Julien Gonzalez, Eric Neau, Christine Delage and Denis Calise performed wet lab experiments. Bénédicte Buffin-Meyer, David Ribes, Joost P. Schanstra, and Jean-loup Bascands designed the study and wrote the manuscript.

### Conflict of interest statement

SSR240612 was a kind gift of Sanofi-Aventis R&D Montpellier-France. The authors declare that the research was conducted in the absence of any commercial or financial relationships that could be construed as a potential conflict of interest.

## Supplementary material

Supplementary Figure 1 **Average expression stability measure of the 16 reference genes**. Transferrin C (Tfrc), Ubiquitin C (Ubc), phosphoglycerate kinase 1 (Pgk1), βActin (Actb), β2microglobulin (B2m), ribosomal protein L13A (Rpl13a), glucuronidaseβ (Gusb), TATA box binding protein (Tbp), importin 8 (Ipo8), RNA polymerase II (RPII), peptidylprolyl isomerase A (Ppia), tyrosine 3-monooxygenase/tryptophan 5-monooxygenase activation zeta polypeptide (Ywhaz), ribosomal RNA 18S (18S), hypoxanthine–guanine phosphoribosyltransferase (Hprt), glyceraldehyde-3-phosphate dehydrogenase (Gapdh), hydroxylmethylbilane synthase (Hmbs).

Supplementary Table 1 **Symbol, Name, and ID gene of the 96 genes present on the Low density Array**.
