# Supplementary figures and images for: Kinin B1 receptor antagonism is equally efficient as angiotensin receptor 1 antagonism in reducing renal fibrosis in experimental obstructive nephropathy, but is not additive
Source: Front Pharmacol. 2015 Feb 2;6:8. doi: 10.3389/fphar.2015.00008 (PMC4313587; doi:10.3389/fphar.2015.00008)

## Slide 1
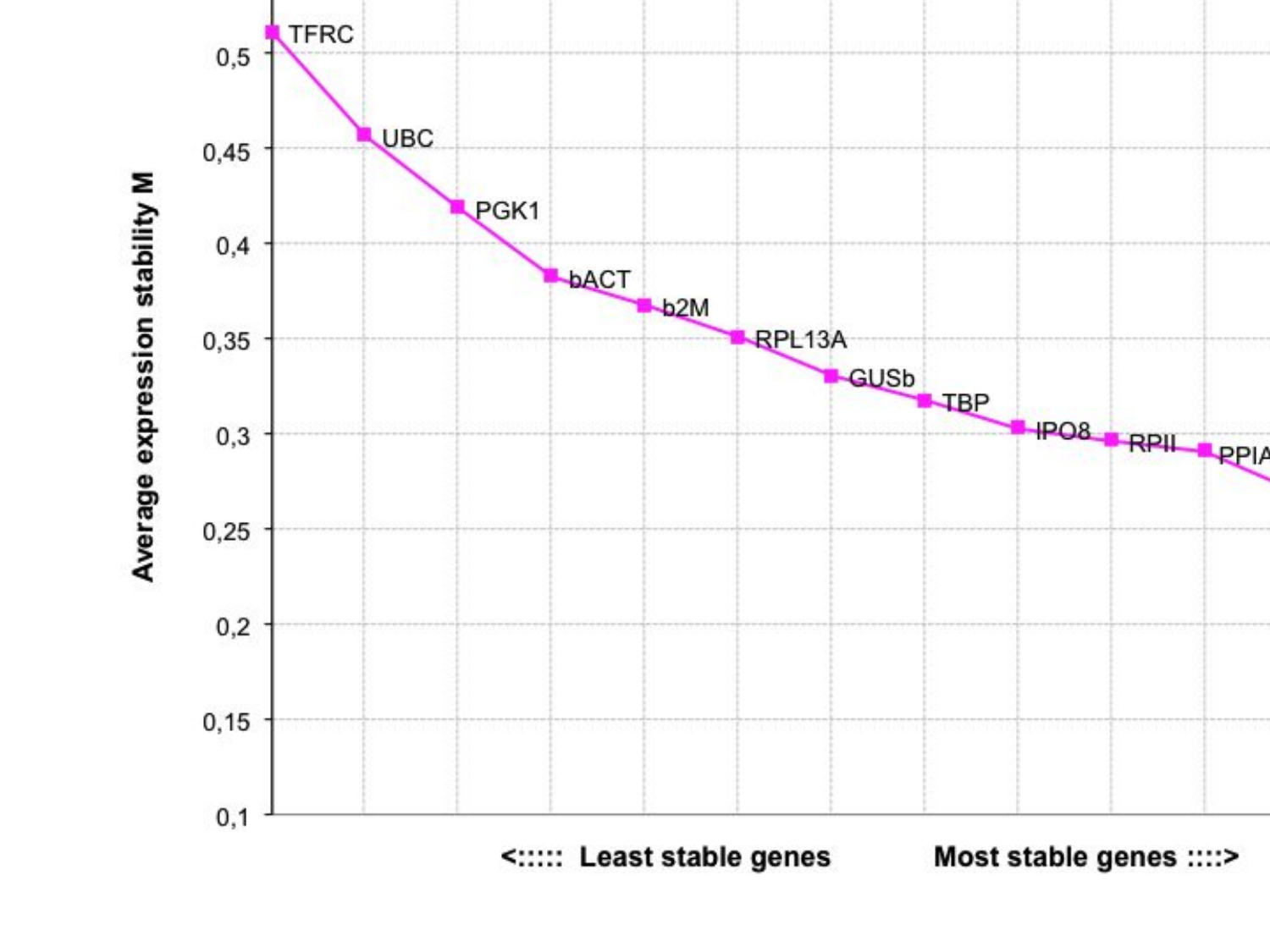

Suppl Figure 1

Supplement: Supplementary Figure 1 — Average expression stability measure of the 16 reference genes. Transferrin C (Tfrc), Ubiquitin C (Ubc), phosphoglycerate kinase 1 (Pgk1), βActin (Actb), β2microglobulin (B2m), ribosomal protein L13A (Rpl13a), glucuronidaseβ (Gusb), TATA box binding protein (Tbp), importin 8 (Ipo8), RNA polymerase II (RPII), peptidylprolyl isomerase A (Ppia), tyrosine 3-monooxygenase/tryptophan 5-monooxygenase activation zeta polypeptide (Ywhaz), ribosomal RNA 18S (18S), hypoxanthine–guanine phosphoribosyltransferase (Hprt), glyceraldehyde-3-phosphate dehydrogenase (Gapdh), hydroxylmethylbilane synthase (Hmbs). [file Presentation1.PPT]
